# Supplementary material for: A Leopard Cannot Change Its Spots: Unexpected Products from the Vilsmeier Reaction on 5,10,15-Tritolylcorrole
Source: Molecules. 2020 Aug 6;25(16):3583. doi: 10.3390/molecules25163583 (PMC7464667; doi:10.3390/molecules25163583)
Supplement: Supplementary file 1 [file molecules-25-03583-s001.pdf]

A leopard cannot change its spots: unexpected products from the Vilsmeier reaction on 5,10,15-tritolylcorrole

Fabrizio Caroleo[§], Greta Petrella[§], Lorena Di Zazzo, Sara Nardis, Beatrice Berionni Berna, Daniel O. Cicero and Roberto Paolesse

SUPPLEMENTARY MATERIAL

CONTENTS

MALDI-TOF of 6 2

MALDI-TOF of 7.. 3

MALDI-TOF of 8.. 3

^{1}H NMR of 8 4

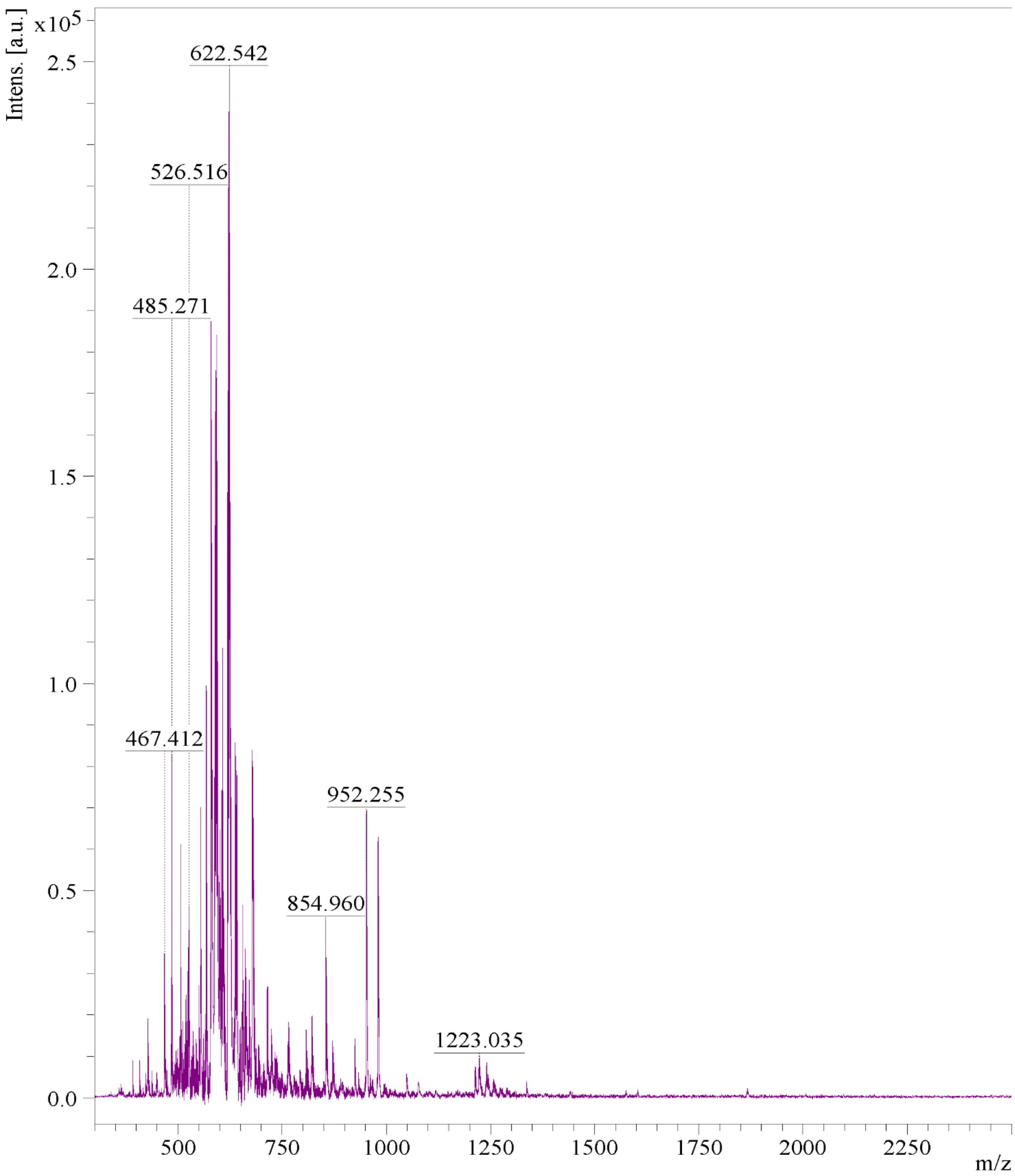

Figure S1. MALDI-TOF of N21,N22-3-formylpropyl-5,10,15-tritolylcorrole **6**.

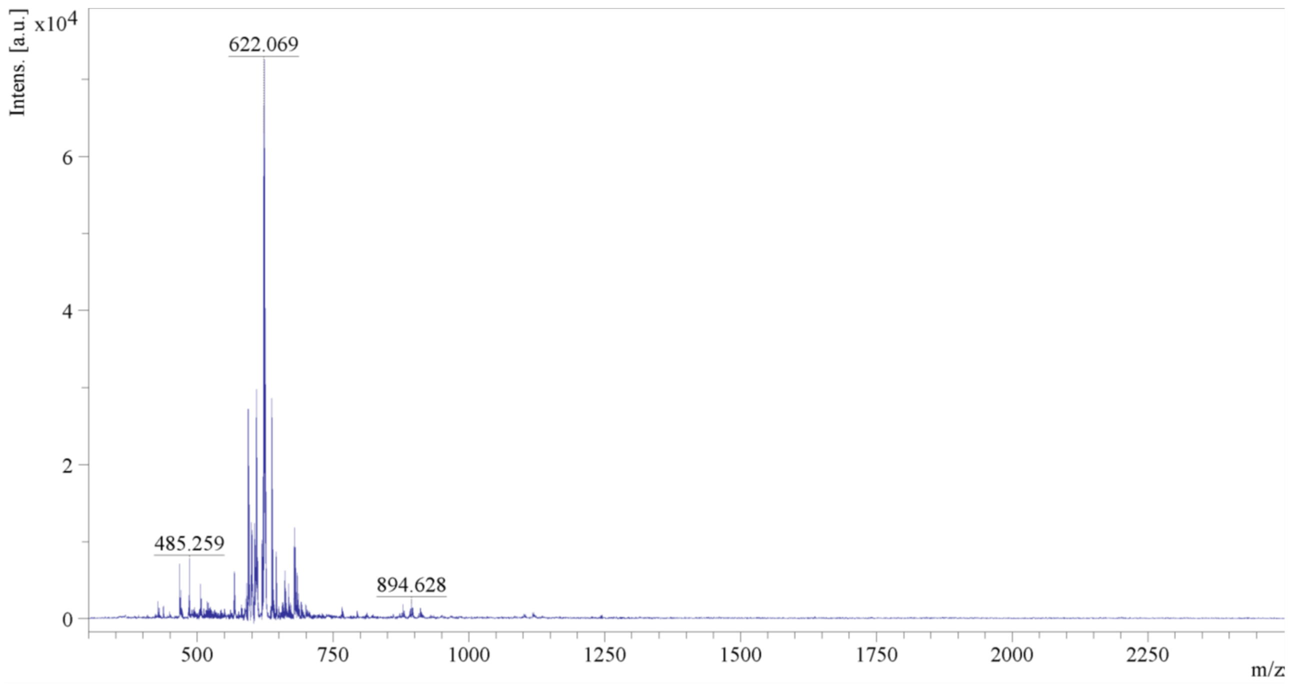

Figure S2. MALDI-TOF of 10-acroleil-5,10,15-tritolyl isocorrole **7**.

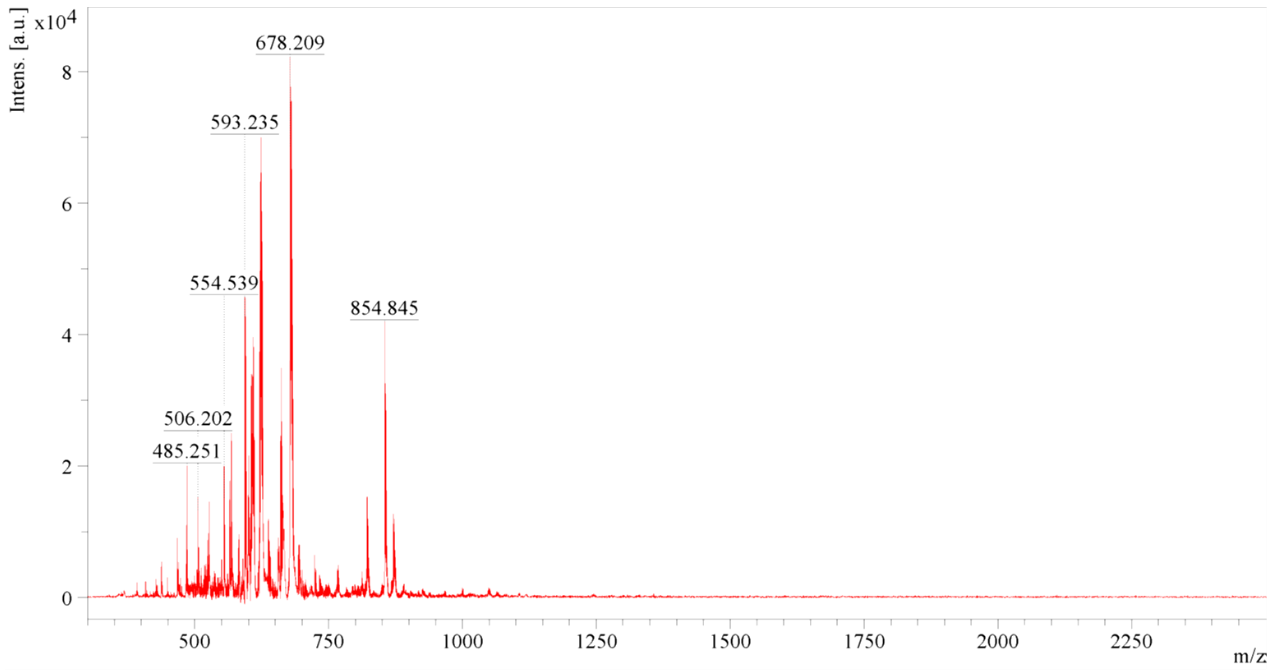

Figure S3. MALDI-TOF of Ni(II)-10-acroleil-5,10,15-tritolyl isocorrole **8**.

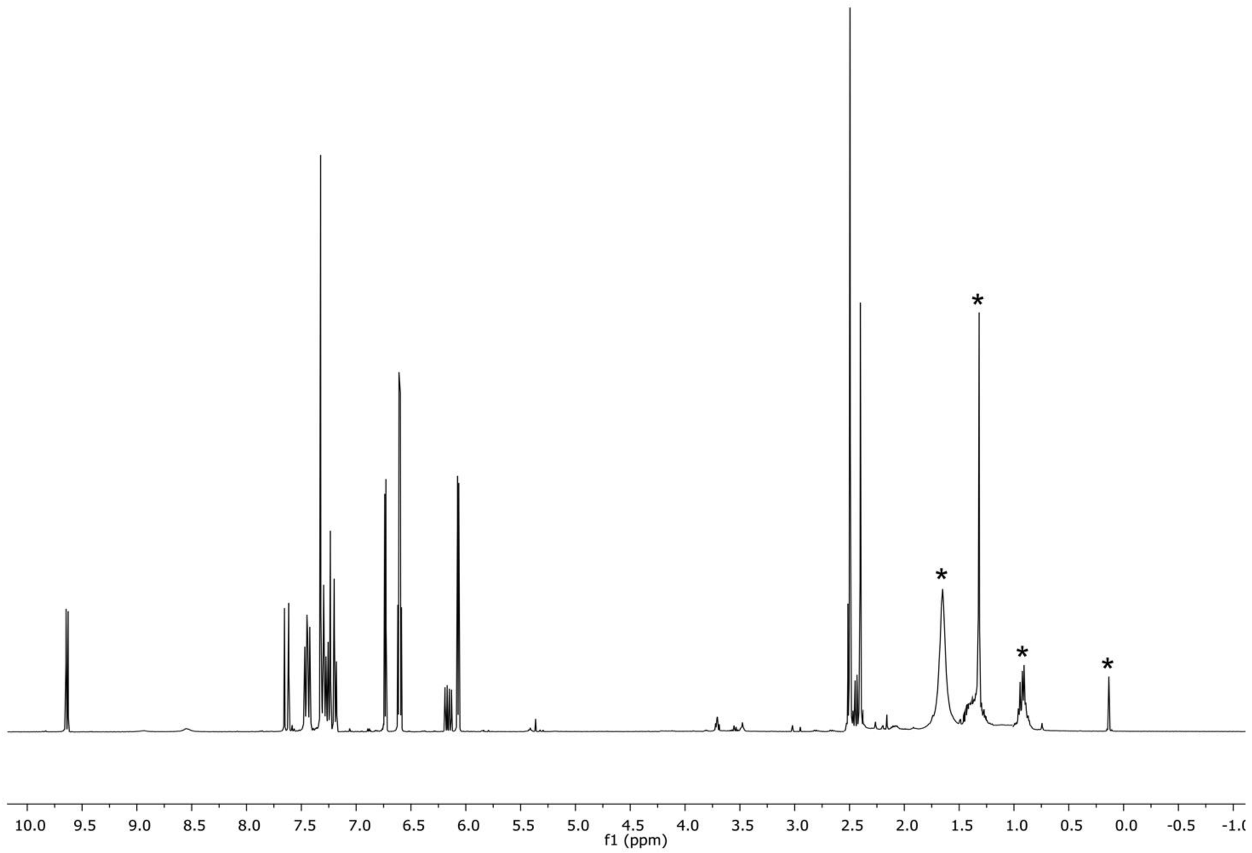

Figure S4. 700 MHz ^{1}H NMR spectrum of Ni(II)-10-acroleil isocorrole **8** in $CDCl_3$ at 25°C.
